# Activation of aldehyde dehydrogenase 2 protects ethanol‐induced osteonecrosis of the femoral head in rat model

**DOI:** 10.1111/cpr.13252

**Published:** 2022-05-14

**Authors:** Xiaoyi Lin, Daoming Zhu, Kaiyang Wang, Pengbo Luo, Gang Rui, Youshui Gao, Fuan Liu, Hongping Yu

**Affiliations:** ^1^ Department of Orthopedic Surgery The First Affiliated Hospital of Xiamen University Xiamen People's Republic of China; ^2^ Department of Medical Imaging The Central Hospital of Enshi Tujia and Miao Autonomous Prefecture Enshi People's Republic of China; ^3^ Department of Spine Surgery Drum Tower Hospital of Nanjing University Medical School Nanjing China; ^4^ Department of Orthopedic Surgery Shanghai Jiao Tong University Affiliated Sixth People's Hospital Shanghai China

## Abstract

**Objectives:**

Osteonecrosis of the femoral head (ONFH) is a devastating disease characterized by destructive bone structures, enlarged adipocyte accumulation and impaired vascularization. The aldehyde dehydrogenase 2 (ALDH 2) is the limiting enzyme for ethanol metabolism with many physiological functions. The aim was investigated the potential protective role of activated ALDH 2 by Alda‐1 for ethanol‐induced ONFH.

**Materials and Methods:**

The ethanol‐induced ONFH in rat was performed to explore the protective of Alda‐1 by various experimental methods. Subsequently, the effect of Alda‐1 and ethanol on the osteogenic and adipogenic differentiation was investigated via multiple cellular and molecular methods. Finally, the effect of Alda‐1 and ethanol on the neo‐vascularization was detected in Human umbilical vein endothelial cells (HUVECs) and ONFH model.

**Results:**

Firstly, radiographical and pathological measurements indicated that alda‐1 protected ethanol‐induced ONFH. Moreover, ethanol significantly inhibited the proliferation and osteogenic differentiation of BMSCs, whereas Alda‐1 could distinctly rescue it by PI3K/AKT signalling. Secondly, ethanol remarkably promoted the lipid vacuoles formation of BMSCs, while Alda‐1 significantly retarded it on BMSCs by AMPK signalling pathway. Finally, ethanol significantly inhibited proliferation and growth factor level resulting in reduced angiogenesis, whereas Alda‐1 could rescue the effect of ethanol. Additionally, Alda‐1 significantly reduced the occurrence of ONFH and promoted vessel number and distribution in alcoholic ONFH.

**Conclusions:**

Alda‐1 activation of ALDH 2 was highly demonstrated to protect ethanol‐induced ONFH by triggering new bone formation, reducing adipogenesis and stimulating vascularization.

## Graphical abstract

Alda‐1 activation of ALDH 2 was highly demonstrated to protect ethanol‐induced ONFH by triggering new bone formation, reducing adipogenesis and stimulating vascularization. Therefore, ALDH 2 may be a potential therapeutic target and small molecule Alda‐1 may be promising pharmacotherapeutic for ONFH in the future.

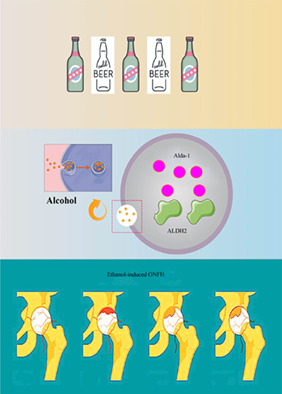

## 1 INTRODUCTION

Osteonecrosis of the femoral head (ONFH) is the death of bone which leads to the collapse of the bone structure of head, joint pain, destructive bone and articular cartilage, and lose of normal functions.^1^ About three quarters of ONFH patients are in the third to sixth decades of their life span.^2^ Although a mass of studies has been extensively explored the aetiology, pathogenesis, diagnosis and therapy of ONFH, no unambiguous consensus of its exact physiological mechanism and origin is been made.^3^, ^4^ Epidemiologic studies show that alcohol consumption is the most common factor for adult patients, but the clear mechanism of how alcohol leading to the blood supply interruption and death of bone cells of femoral head is unclear until now.^5^, ^6^

Chronic and excessive alcohol consumption has become a major health event with rising mortality and morbidity in the whole world, which lead to a vast of abnormal biochemical, physiological and clinical matters that come from the toxic effect of alcohol on human bodies, like liver, gonads, heart, brain, marrow and bone.^7^, ^8^ Alcohol consumption is a vital factor for more than 60 diseases and injures, and leads to about 2.5 million deaths per year (World Health Organization 2011). The detoxifying type of alcohol in human mainly occurs in liver by two enzymatic steps. Firstly, alcohol is quickly converted to acetaldehyde by alcohol dehydrogenase (ADH). Secondly, acetaldehyde is limited metabolized to acetate by the mitochondrial aldehyde dehydrogenase 2 (ALDH 2).^9^, ^10^ Notably, the first step of alcohol metabolism is a reversible reaction, but the second step of alcohol metabolism is a limiting step. Moreover, ALDH 2 is possible the only enzyme for acetaldehyde metabolism.^9^ Therefore, ALDH 2 exerts a vital protective, preventive and therapeutic enzymatic function against alcohol toxicity on human. Such as, chronic alcohol‐induced insulin insensitivity and cardiac dysfunction was alleviated by ALDH 2.^11^ ALDH 2 ameliorates ethanol‐induced atrial fibrillation by detoxifying 4‐HNE.^12^ Active ALDH 2 prevents ethanol‐induced cell death and hepatic steatosis in mice.^13^

Alda‐1 is a small molecule, a chemical chaperone and agonist of ALDH 2, which specifically binds to ALDH 2 and is capable of elevating the catalytic ALDH 2 enzyme activity more than tenfolds.^14^ Accumulative research has indicated that Alda‐1 exerts a lot of protective effect by activating ALDH 2. For example, Alda‐1 accelerates cutaneous wound healing by Akt/GSK‐3β/β‐Catenin signalling pathway via activating ALDH 2.^15^ Alda‐1 activation of ALDH 2 shows the therapeutic effect in myocardial ischaemia–reperfusion injury.^16^ Activation of ALDH 2 by Alda‐1 decreases lung oedema, alveolar epithelial tissue permeability and lung inflammatory via accelerating the clearance of aldehydes.^17^ Moreover, Alda‐1 protects alcohol‐induced DNA damage in oesophagus via activation of ALDH 2.^18^ Alda‐1 pharmacologically activating ALDH 2 prevents and protects ethanol‐induced hepatic cell death and steatosis by reactivating transcription factors and upregulating fatty acid oxidation enzymes in mice.^13^ Alda‐1 pharmacological activation of ALDH 2 effectively reducing the maintenance and acquisition of chronic alcohol intake in rats.^19^ Therefore, we hypothesized that Alda‐1 has a potential protective effect on alcohol‐induced ONFH by activating ALDH 2. In the present study, our results indicated that Alda‐1 prevented alcohol‐induced decreased bone formation and vascularization, and increased adipogenesis in alcohol‐induced rat ONFH model.

## 2 MATERIALS AND METHODS

### 2.1 Animal model

With the approval of the Animal Committee at the The First Affiliated Hospital of Xiamen University, 38‐week male Sprague–Dawley rats were performed to build the ethanol‐induced ONFH experiment. The whole rats were randomly divided into the control group, ethanol (ET) group and ET + Alda‐1 (50 mg/kg, i.p.) group. The normal Lieber‐DeCarli liquid diet (without ethanol) was adapted for rats 1 week. Then, the ethanol‐containing Lieber‐DeCarli liquid diet was fed for the ET group and the ET + Alda‐1 group, but the ET + Alda‐1 group was intraperitoneally injected with Alda‐1 (50 mg/kg/d). At the same time, the control group was fed with the normal Lieber‐DeCarli liquid diet. The diet was refreshed every day and all rats had free availablality to food. In order to reflect the dynamic new bone formation, fluorescence staining (20 mg/kg tetracycline, 10 mg/kg calcein‐AM and 30 mg/kg alizarin red S) (Aladdin) was intraperitoneally injected at week 0, 2 and 4.

### 2.2 Micro‐CT scanning

Microfil (MV‐122, FlowTech) was performed to visualize the vasculatures in the femoral heads of rats. After general anaesthesia, the left ventricles of all rats were perfused with 80 ml heparinized saline and 25 ml MicroFil. In order to sufficiently polymerize the MicroFil, all rats were stored in a refrigerator at 4°C overnight. Then, the bilateral femoral heads of all rats were harvested. Then all samples were scanned with the micro‐CT scanner (SkyCcan 1176) after 24 h fixed in 4% paraformaldehyde. The DataViewer software was used to reconstructed the images. The CTan software was performed to calculate the parameters of the femoral heads, including trabecular bone volume fraction (BV/TV), trabecular thickness (Tb.Th), bone mineral density (BMD) and trabecular number (Tb.N). Ten percent EDTA was performed to decalcified the femoral heads for 1 month. Finally, all heads all scanned again by the scanner for visualization of the vasculatures, and the CTvol software was used to reconstructed the 3D shapes of vasculatures in femoral heads.

### 2.3 Histological staining

The decalcified femoral heads were embedded in paraffin, and cut into 5 μm slices in coronal position. Then the sections were stained by haematoxylin and eosin (H&E) for pathological observation of osteonecrosis in the femoral heads. Immunohistochemical staining (IHC) was performed to compare the expression of collagen I (COL I, 1:100, CST, USA) and osteocalcin (OCN, 1:100, CST) in the femoral heads. All images were captured by a microscope (LEICA, German).

### 2.4 Cell culture

The femurs and tibias of 4‐week‐old SD rats were performed to obtained bone mesenchymal stem cells (BMSCs) according to the previous reports.^20^, ^21^ α‐minimum essential medium (α‐MEM) (Gibco, USA) with 10% foetal bovine serum (FBS, Gbico) and 1% penicillin/streptomycin (Gbico) were used to incubated with BMSCs in a humidified atmosphere at 37°C with 5% CO_2_. The third to the seventh passage of BMSCs were performed for the whole research. Human umbilical vein endothelial cells (HUVECs) were purchased from Procell (Wuhan, China) and incubated with endothelial cell medium (ECM, Gibco).

### 2.5 Cell proliferation assay

Cell Counting Kit‐8 assay (CCK 8, Solarbio, China) was used to evaluate the effect of ethanol (100 mM) and alda‐1 (20 μM) on BMSCs and HUVECs. In brief, 5 × 10^3^ cells in 100 μl medium per well were seeded in 96‐well plates. According to the protocol, every well was added with 10 μl CCK 8 solution and 90 μl medium. Then the absorbance values of supernatants were tested at 450 nm after incubation of the plates in 37°C for 2 h.

### 2.6 Osteogenic differentiation assay

Alizarin red staining (ARS, Solarbio) and alkaline phosphatase (ALP, Solarbio) staining were performed to investigate the effect of ethanol (100 mM), alda‐1 (20 μM) and the PI3K antagonis LY294002 (500 nM) on the osteogenic differentiation of BMSCs. Briefly, 1 × 10^5^ cells in 500 μl medium per well were seeded in 24‐well plates. Then osteogenic medium (Cyagen, China) was added to every well for inducing osteogenic differentiation when the confluence of cells was 80%, and the osteogenic medium was refreshed by every 2 days. The ARS and ALP staining were conducted at day 21 and day 7 according to the protocols, respectively, and a Leica microscope was used to capture all images.

### 2.7 Immunofluorescent staining

The effect of ethanol (100 mM), alda‐1 (20 μM) and the PI3K antagonis LY294002 (500 nM) on the change of osteogenic‐associated markers BMSCs was explored by immunofluorescent staining (IF). BMSCs were seeded in the middle spot of 35‐mm confocal dishes for 48 h. Then, cells were washed, fixed, permeabilized, blocked and incubated with the primary antibodies (COL I and OCN, 1:100, CST, USA). Then, cells were washed and incubated with the secondary antibodies (1:500, CST), DAPI and phalloidin. Finally, a Leica microscope was used to capture images.

### 2.8 Adipogenic differentiation assay

Oil red O staining (Solarbio) was performed to investigate the effect of ethanol (100 mM), alda‐1 (20 μM) and the AMPK antagonis DMP (10 μM) on the adipogenic differentiation of BMSCs. In briefly, each well of six‐well plates was seeded with 2 × 10^5^ cells in 2 ml medium. Then adipogenic medium (Cyagen, China) was added to every well for inducing adipogenic differentiation when the confluence of cells was 80%, and the adipogenic medium A and B were refreshed by the protocol. Oil red staining was performed at day 21.

### 2.9 Cell migration assay

The effect of ethanol (100 mM), alda‐1 (20 μM) and the AMPK antagonis DMP (10 μM) on the migration of HUVECs was investigated by wound healing assay and transwell assay. As for wound healing assay, each well of sixwell plates was seeded with 2 × 10^5^ cells in 2 ml medium. Then a sterile pipette tip was used to scratch the confluent cell monolayer, and different treatment of medium was added to each well. Finally, images were captured at the specific time‐point and Image J was performed to measure the rates of wound healing. For transwell assay, the upper chambers of the 24‐well transwell plate were seeded with 2 × 10^4^ cells in 200 μl low concentration FBS medium, while the lower chambers were added with 700 μl high concentration FBS medium. After 24 h, cells on the upper surfaces were erased after fixed. Then crystal violet was performed to stain cells on the lower surface. Finally, 6 random areas of the lower surface were chosen and counted total cells by a microscope.

### 2.10 Angiogenesis assay

Tube formation assay was performed to the effect of ethanol (100 mM), alda‐1 (20 μM) and the AMPK antagonis DMP (10 μM) on the vascularization of HUVEC. Briefly, each well of the 96‐well plate was added with 50 μl Matrigel (BD Bioscience, USA), and transferred to the incubator 30 min for totally gelatinization. A Leica phase‐contrast microscope was used to capture the tube formation images at 6 h, and Image J was performed to count the number of nodes and complete capillaries.

### 2.11 Quantitative polymerase chain reaction

Quantitative polymerase chain reaction (QPCR) was performed to the expression level of relative mRNA in BMSCs or HUVECs. After cells were incubated in sixwell plates with different treatment of medium for 48 h, total mRNA of cells was obtained following the protocols (EZBioscience, USA). Equal purified RNA was used for the reverse transcriptase reaction, and the SYBR premix ex taq kit (EZBioscience) was used for QPCR. GAPDH was used as the internal gene. The primers were as Table S1.

### 2.12 Western blot assay

Western blot (WB) assay was performed to investigate the relative target protein level in BMSCs or HUVECs. Briefly, cells were incubated in sixwell plates with different treatment of medium for specific time, cells were lysed by RIPA lysis buffer (1 mM PMSF, Beyotime, China) for the extracting the total. Then the protein concentration was tested, equal protein was electrophoresed, transferred, blocked and incubated with the primary antibodies for GAPDH, COL I, OCN, AKT, p‐AKT, AMPK and p‐AMPK (1:1000, CST). Next, PVDF membrane was washed and incubated with the secondary antibodies (1:5000). Finally, the membrane was reacted with ECL kit (Thermo Scientific, USA), and the signal was quantified by a scanning densitometry.

### 2.13 ELISA

The absolute quantification VEGF content in HUVECs was detected by a VEGF ELISA kit (Neobioscience, China). The liquid supernatant of cells was obtained after HUVECs were incubated with different treatment for 48 h. The supernatant was centrifuged, diluted and detected of VEGF following the manufacturer's protocol. The absorbance values were gained at 450 nm used for calculating the VEGF level according to the standard curve.

### 2.14 Statistical analysis

All data were shown in means ± SEM. The SPSS 20.0 (IBM, USA) was used to analyse the statistically difference by one‐way ANOVA or Student's test. *p < .05 was considered to have statistical significance.

## 3 RESULTS

### 3.1 Alda‐1 protected ethanol‐induced ONFH in rats

**FIGURE 1:**
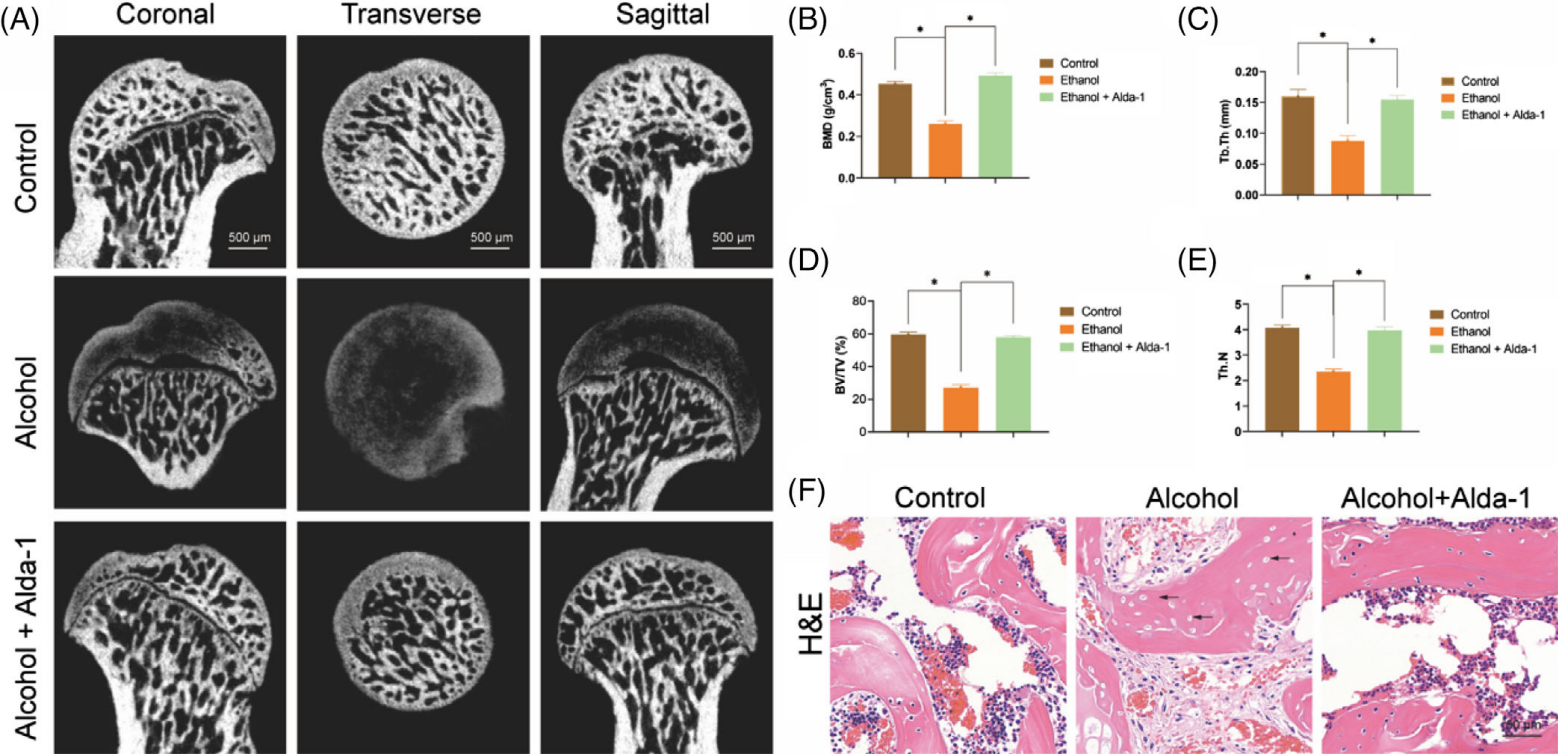
Alda‐1 protected ethanol‐induced ONFH in rats. (A) Micro‐CT scanning images of the femoral heads. (B–E) BMD, Tb.Th, BV/TV and Tb.N were calculated by reconstructed CT images. Results were means ± SEM of five specimen. (F) H&E staining of the femoral heads. Empty lacunae in the subchondral trabeculae were marked by black arrows

Alda‐1 activation of ALDH2 exerts a lot of protective effect in alcoholic animal models, so we hypothesis that Alda‐1 might also have protective effect in ethanol‐induced ONFH. It is consistent with our hypothesis that intraperitoneal injection of Alda‐1 alleviated ethanol‐induced ONFH in rat model because of the following reasons and evidences. All group rats had the gradual and similar weight gain in the whole experiment (Figure S1). Firstly, micro‐CT scanning of the rat femoral heads indicated that there were remarkably bone defect and signs of osteonecrosis in the ethanol group (8 of 10 rats) (Figure 1A). However, only one rat of the ethanol+Alda‐1 group exerted mild signs of bone defect and osteonecrosis, and there was no signs of bone defects or osteonecrosis in the Control group (Figure 1A). Secondly, the numerical analysis of the micro‐CT scanning further indicated that the important bone parameters of the ethanol group (BMD, BV/TV, Tb.N and Tb.Th) were significantly reduced, as compared with those of in the Control group (Figure 1B–E). Notably, these bone parameters were significantly upregulated in the femoral heads of the ethanol+Alda‐1 group, as compared with the ethanol group. Thirdly, H&E staining proved the protective effect of Alda‐1 on alcohol‐induced necrosis of femoral head. As shown in Figure 1F, there were large pyknosis of nuclei in the trabeculae, diffused empty lacunae and bone marrow haematopoietic cellular debris in the medullary spaces in the ethanol group. On the contrary, there were no apparent histopathological osteonecrosis signs in the ethanol+Alda‐1 group or the control group, except a bit of marginal pathological signs in the ethanol + Alda‐1 group (Figure 1F).

**FIGURE 2:**
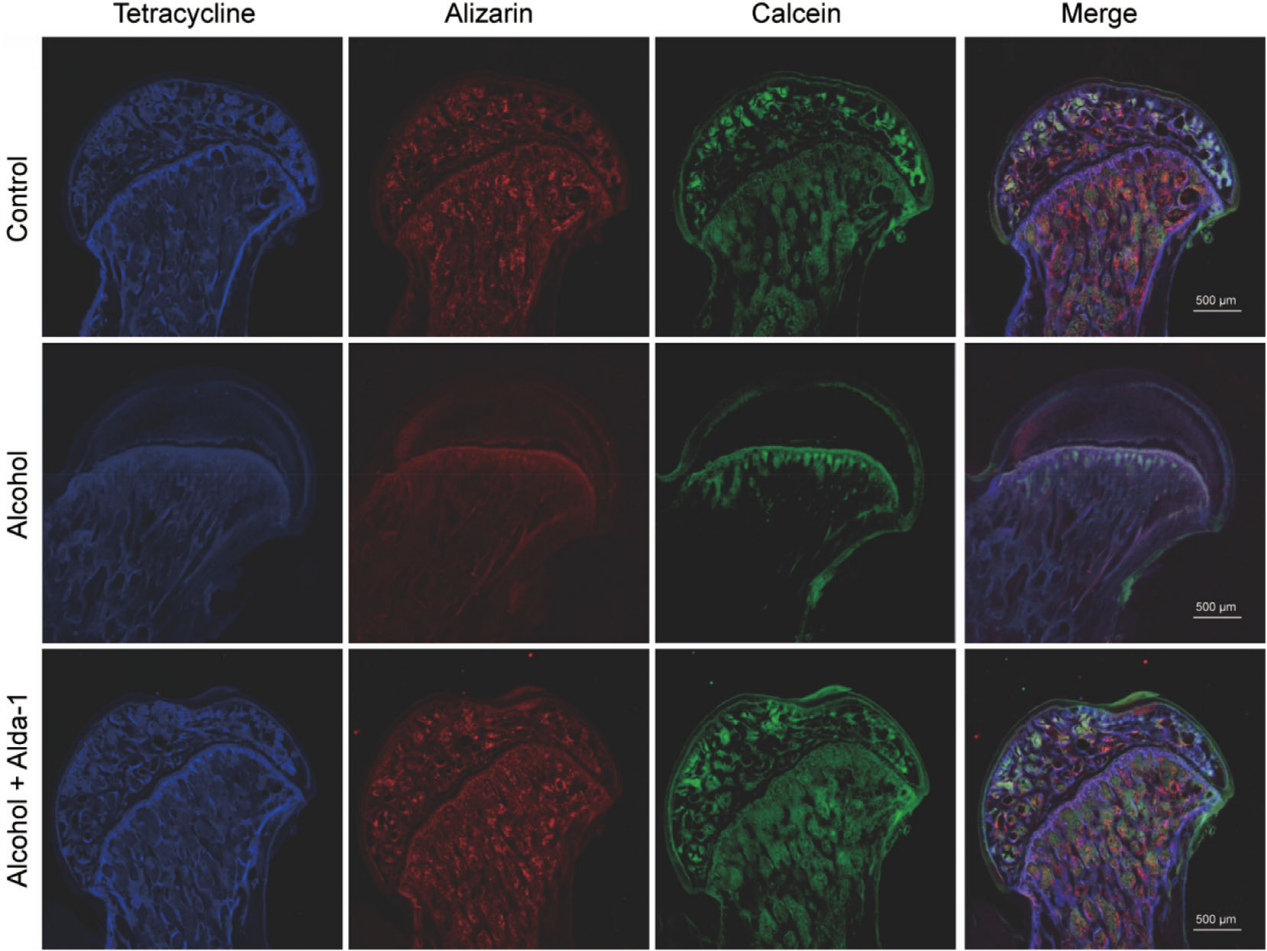
The protective effects of Alda‐1 against ethanol‐induced ONFH in the rat model

Finally, fluorescence labelling was performed to reflect the dynamic new bone formation and mineralization of the femoral heads in rats. The subchondral femoral head trabeculae of the control group were all deposited with tetracycline (blue), Alizarin Red (red) and calcein (green) (Figure 2). On the contrary, there were significantly reduced tetracycline, Alizarin Red and calcein deposition in the subchondral femoral head trabeculae of the ethanol group, manifesting reduced new bone formation and upregulated bone resorption (Figure 2). However, a large area of the subchondral femoral head trabeculae in the ethanol+Alda‐1 group were labelled tetracycline, Alizarin Red and calcein compared with the ethanol group, indicating upregulated new bone formation and reduced bone resorption of the femoral head in the ethanol+Alda‐1 group (Figure 2).

### 3.2 Alda‐1 alleviated the suppressive osteogenesis of ethanol on BMSCs by PI3K/AKT signalling pathway

**FIGURE 3:**
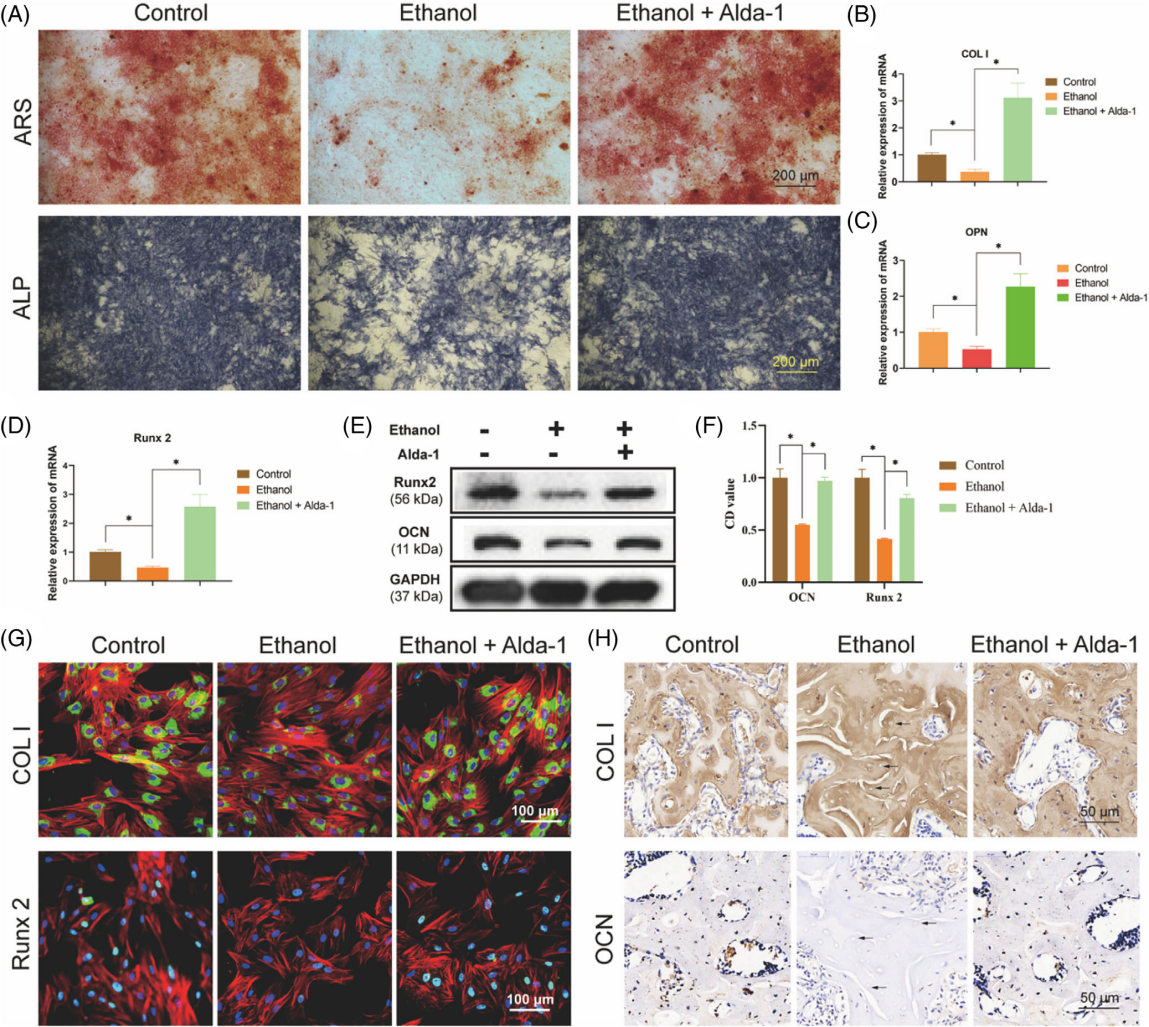
Alda‐1 alleviated the suppressive osteogenesis of ethanol on BMSCs. (A) The ARS and ALP staining of BMSCs. (B–D) The mRNA level of COL I, OPN and Runx2 in BMSCs. Results were means ± SEM of four independent experiments in triplicate. (E, F) The protein level of Runx2 and OCN in BMSCs. Results were means ± SEM of four independent experiment. (G) Immunofluorescence staining of COL I and Runx2 in BMSCs. (H) Immunohistochemical staining of COL I and OCN

To begin with, we performed CCK‐8 assay to evaluate the effect of ethanol and alda‐1 on the proliferation of BMSCs. The results indicated that ethanol remarkably inhibited the proliferation of BMSCs compared with the control group, while this inhibition of ethanol was substantially reversed by co‐treatment with Alda‐1 in the athanol+Alda‐1 group (Figure S2). Subsequently, the ARS assay indicated that ethanol significantly inhibited the osteogenic differentiation of BMSCs as there was remarkably reduced mineralization nodes and calcium deposition in the ethanol group, as compared with the control group (Figure 3A). However, the ethanol‐induced, reduced osteogenesis of BMSCs was restored by co‐treatment of Alda‐1 in the ethanol+Alda‐1 group (Figure 3A). The ALP staining got the similar results, i.e., Alda‐1 could restore the ethanol‐induced inhibition of the ALP activity (Figure 3A). Then, the RT‐PCR results revealed that ethanol distinctly reduced the gene expression of the osteogenic‐associated markers, COL I, OPN and Runx2. However, Alda‐1 treatment could prevent the inhibition of ethanol (Figure 3B–D). We also performed western blot to investigate the expression of osteogenic‐associated protein. The protein expression of COL I and Runx2 was significantly reduced by ethanol treatment for 48 h, while Alda‐1 could also antagonize the inhibition of ethanol on BMSCs (Figure 3E,F). Subsequently, the direct visualization and quantification of osteogenic‐associated markers in BMSCs were tested by IF. As shown in Figure 3G, the fluorescence intensity of COL I and Runx2 were significantly decreased in the ethanol group as compared with the control group, whereas co‐treatment Alda‐1 and ethanol could reverse ethanol‐induced inhibition on BMSCs. Finally, IHC of COL I and OCN were performed to evaluate osteogenesis effect of ethanol and Alda‐1 on the femoral head of rats. The ethanol group had more weaker staining intensity of COL I and OCN as compared with the control group, indicating reduced osteogenic activity and new bone formation (Figure 3H). While Alda‐1 restored the osteogenic activity and new bone formation in the ethanol+Alda‐1 group (Figure 3H).

**FIGURE 4:**
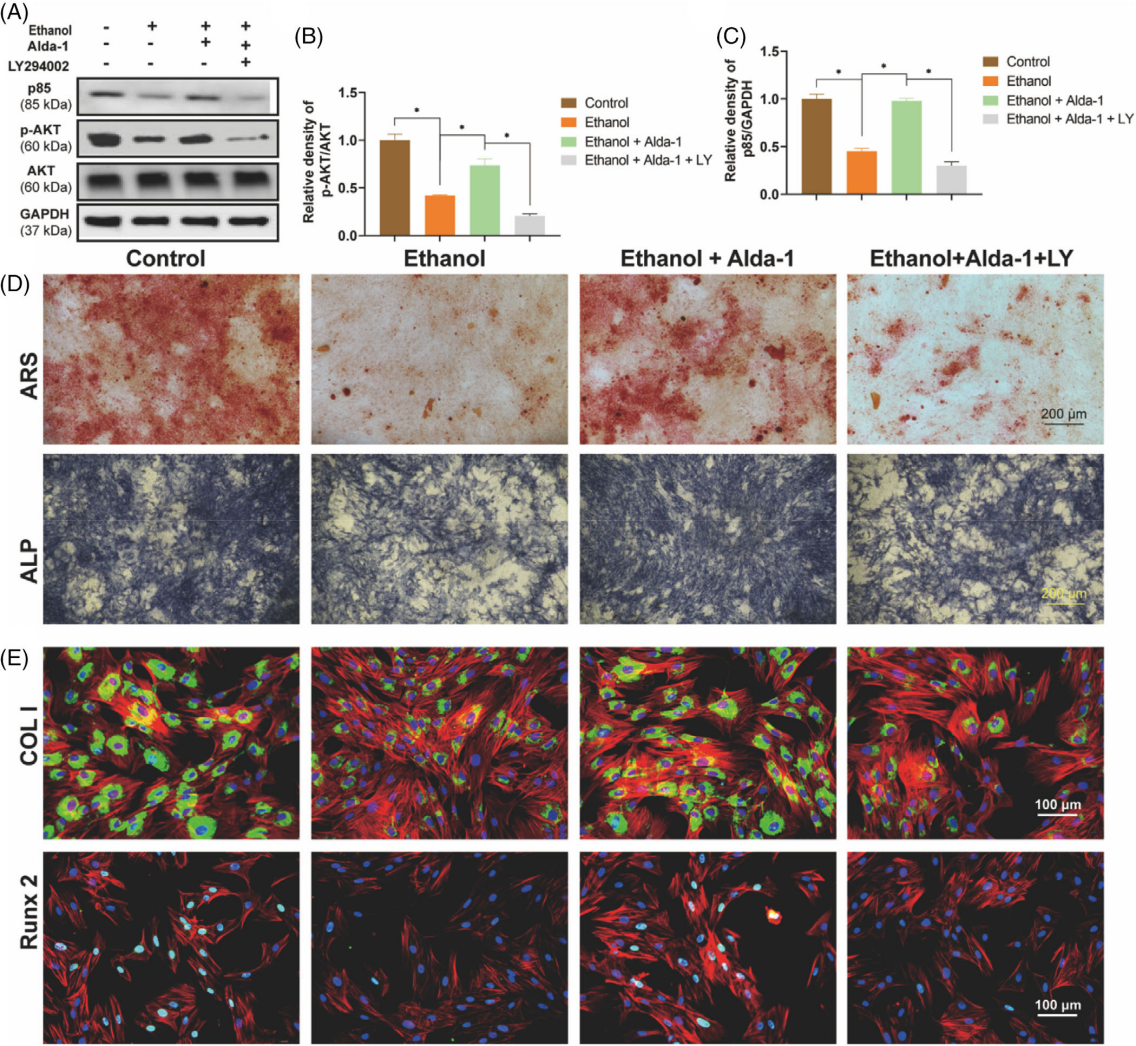
Alda‐1 abolished the ethanol‐induced inhibition on osteogenesis via PI3K/AKT pathway. (A–C) p85 and p‐AKT were decreased by ethanol and increased by Alda‐1 in BMSCs. Results were means ± SEM of four independent experiments. (D) The ARS and ALP staining of BMSCs. (E) Immunofluorescence staining of COL I and Runx2 in BMSCs

Accumulative research displays that PI3K/AKT signalling pathway exerts key role in osteogenesis of BMSCs, so we explored the role of PI3K/AKT pathway in this study. The western blot results showed that the expression level of p85 and phosphorylated AKT were obviously reduced by ethanol treatment, the expression level of GAPDH and total AKT was not changed (Figure 4A–C). Notably, Alda‐1 could rescue the expression level of p85 and p‐AKT (Figure 4A–C). Moreover, we performed the selective antagonist of PI3K/AKT pathway LY294002 to clarify that PI3K/AKT pathway was actually involved in ethanol‐induced inhibition of osteogenesis. The increased expression of p85 and p‐AKT when BMSCs were co‐treated with ethanol and Alda‐1 was reduced by LY294002 treatment (Figure 4A–C). What is more, the involvement of PI3K/AKT pathway was further demonstrated by the morphological performance in vitro. The protective effect of Alda‐1 on ethanol‐induced inhibitive mineralization nodes, calcium deposition, and ALP activity were dramatically abolished by LY294002 co‐treatment (Figure 4D). The IF staining of COL I and Runx2 got the similar results (Figure 4E). Taken together, these results indicated that the Alda‐1 alleviated the ethanol‐induced ONFH by PI3K/AKT signalling pathway.

### 3.3 Alda‐1 retarded the ethanol‐induced adipogenesis on BMSCs by AMPK signalling pathway

**FIGURE 5:**
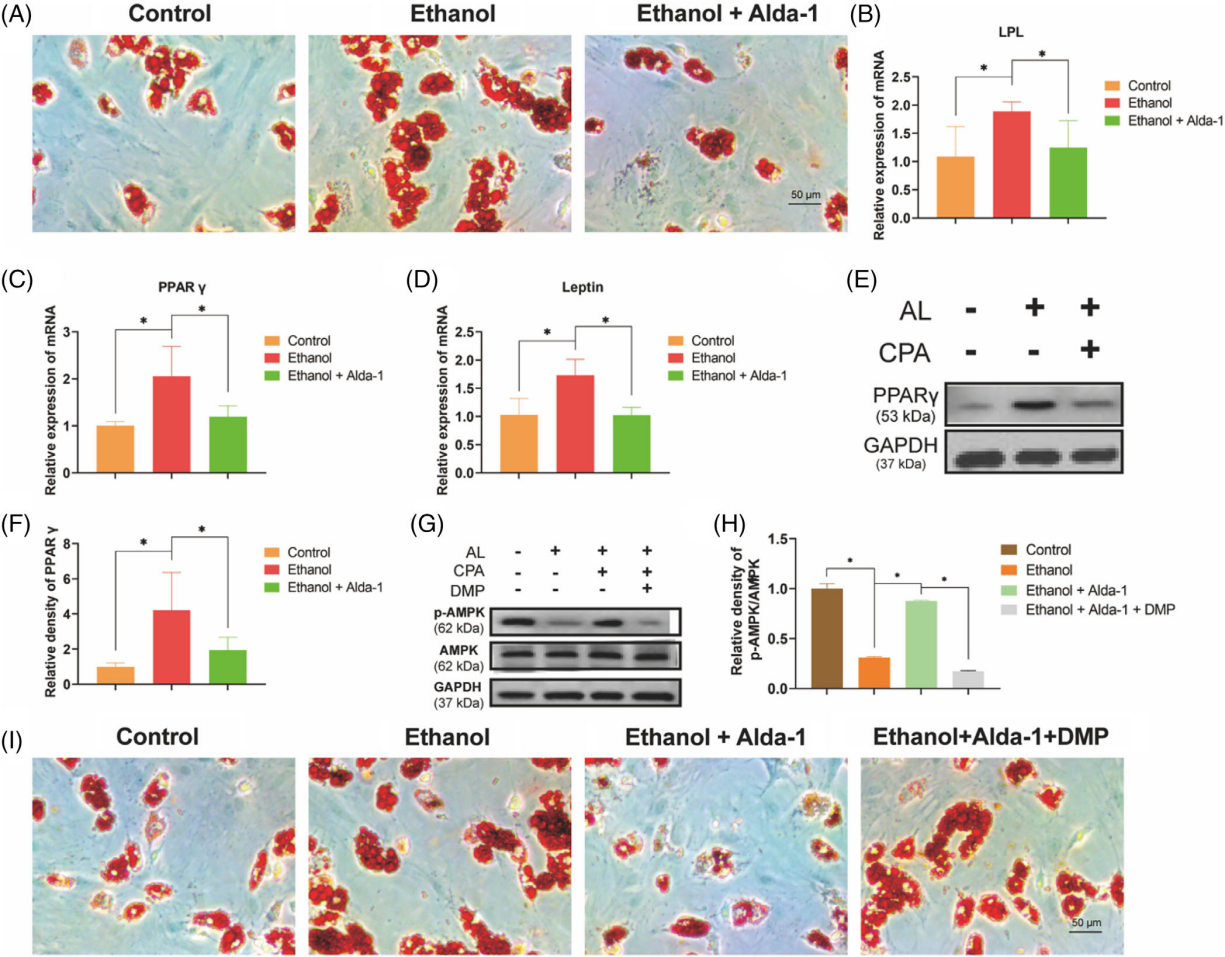
Alda‐1 retarded the ethanol‐induced adipogenesis on BMSCs. (A) The oil red O staining of BMSCs. (B–D) The mRNA level of LPL, PPARγ and leptin in BMSCs. Results were means ± SEM of four independent experiments in triplicate. (E, F) The protein level of PPARγ in BMSCs. Results were means ± SEM of four independent experiment. (G, H) The protein level of p‐AMPK in BMSCs. Results were means ± SEM of four independent experiment. (I) The oil red O staining of BMSCs

Adipogenesis and osteogenesis are reciprocal processes, and fat accumulation is a vital pathogenic factor and pathological process of ONFH. Therefore, we explored the role of ethanol and Alda‐1 on the adipogenesis of BMSCs. The oil red staining indicated that a world of lipid vacuoles formation in the ethanol group as compared with the control group, suggesting the powerful promotive adipogenesis of ethanol (Figure 5A). However, Alda‐1 could distinctly retard ethanol‐induced adipogenesis in the ethanol+Alda‐1 group (Figure 5A). Subsequently, the protein and gene expression level of adipokines were detected by RT‐PCR and western blot. Ethanol treatment obviously increased the gene expression level of lipoprotein lipase (LPL), PPARγ and leptin as compared with the control group, while Alda‐1 distinctly retarded this promotive effect (Figure 5B–D). In addition, the protein expression level of PPARγ was also increased by ethanol treatment, whereas Alda‐1 co‐treatment significantly abolished this upregulation (Figure 5E,F).

The mechanism of ethanol and Alda‐1 on the adipogenesis of BMSCs was further studied. The AMPK pathway is a classic signalling in fat metabolism. The protein expression level of phosphorylated AMPK was significantly reduced by ethanol treatment as compared with the control group, while Alda‐1 obviously reversed the ethanol‐induced inhibition of p‐AMPK in BMSCs (Figure 5G,H).

Moreover, we performed the selective antagonist of AMPK pathway Dorsomorphin (DMP) to clarify that AMPK pathway was actually involved in ethanol‐induced inhibition of adipogenesis. The increased expression of p‐AMPK when BMSCs were co‐treated with ethanol and Alda‐1 was reduced by DMP treatment (Figure 5G,H). What is more, the involvement of AMPK pathway was further demonstrated by the morphological performance in vitro. The inhibitive effect of Alda‐1 on ethanol‐induced adipogenesis were dramatically abolished by DMP co‐treatment (Figure 5I). Taken together, these results indicated that Alda‐1 retarded the ethanol‐induced adipogenesis by AMPK pathway.

### 3.4 Alda‐1 rescued the ethanol‐induced destruction of vascularization

**FIGURE 6:**
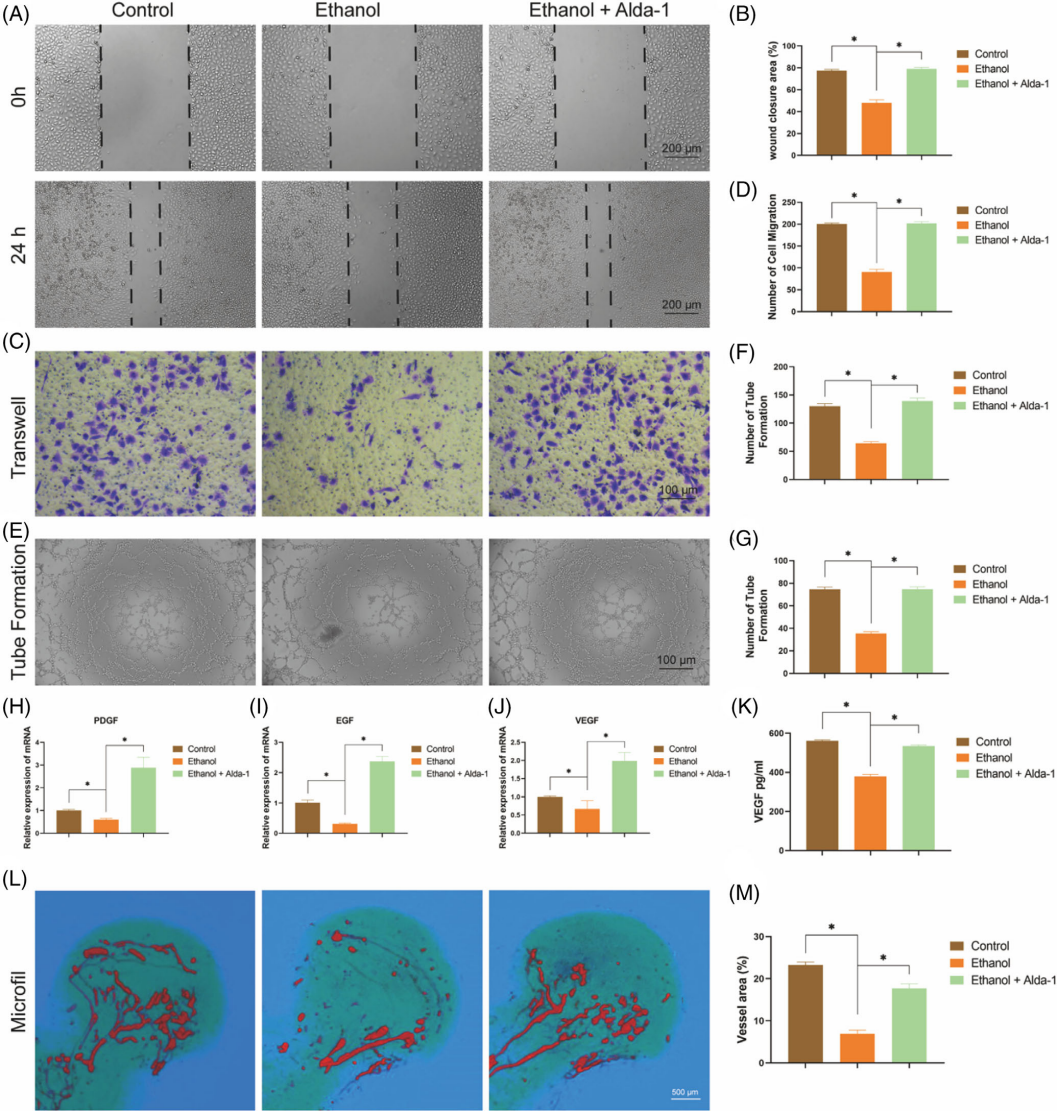
Alda‐1 rescued the ethanol‐induced destruction of vascularization. (A, B) The wound healing assay of HUVECs. Results were means ± SEM of four independent experiments in duplicate. (C, D) The transwell assay of HUVECs. Results were means ± SEM of four independent experiments in duplicate. (E–G) The tube formation assay of HUVECs. Results were means ± SEM of four independent experiments in duplicate. (H–J) The mRNA level of PDGF, EGF and VEGF in HUVECs. Results were means ± SEM of four independent experiments in triplicate. (K) The protein level of VEGF in HUVECs. Results were means ± SEM of four independent experiment in duplicate. (L, M) The 3D reconstruction images of the MicroFil assay of the femoral head. Results were means ± SEM of five femoral heads

Firstly, we performed CCK‐8 assay to evaluate the effect of ethanol and alda‐1 on the proliferation of HUVECs. The results indicated that ethanol remarkably inhibited the proliferation of HUVECs compared with the control group, while this inhibition of ethanol was substantially reversed by co‐treatment with Alda‐1 in the athanol+Alda‐1 group (Figure S3). The primary stage of vascularization is the migration of endothelial cells, so the effect of ethanol and Alda‐1 on the migration of HUVECs was investigated by wound healing assay and transwell assay. Ethanol significantly inhibited the migration of HUVECs in turn resulting in lower wound closure area as compare with the control group, while Alda‐1 could reverse this effect of ethanol (Figure 6A,B). The transwell assay got the similar results with wound healing assay, i.e., ethanol treatment significantly inhibited the number of migrations of HUVECs, whereas Alda‐1 could reverse the inhibitory effect (Figure 6C,D). For the next, tube formation assay was performed to mimic the vascularization. The ethanol group had fewer loop structures and branch points as compared with the control group, while ethanol‐induced inhibition of tube formation was rescued by Alda‐1 co‐treatment (Figure 6E–G). Then, the angiogenic‐associated gene expression of PDGF, EGF and VEGF was detected by RT‐PCR. These expression levels were distinctly decreased by ethanol treatment, while the inhibitive effect of ethanol was reversed by Alda‐1 treatment (Figure 6H–J). The protein expression of VEGF was also detected by ELISA. As shown in Figure 6K, ethanol treatment remarkably reduced the level of VEGF, while Alda‐1 could rescue it.

Finally, micro‐CT scan of the microfil perfusion was performed to evaluate angiogenic effect of ethanol and Alda‐1 on the femoral head of rats. The femoral head of the control group exerted abundant and intact vascular morphology and distribution. On the contrary, there was almost on complete vascular morphology, and the area of vascular distribution was very small in the ethanol group. However, the ethanol+Alda‐1 group had much more intact vascular morphology and distribution as compared with the ethanol group, indicating increased vascularization (Figure 6L,M). All in all, Alda‐1 exerted protective effect against the toxic effect of ethanol on vascularization leading to protecting ethanol‐induced ONFH.

## 4 DISCUSSION

ONFH is a devastating disease and been called the deathless cancer, because it seems like not fatal, but it will cause long‐term pain, walking disorders in patients, or even unable to walk normally, whole life needs people to take care of. There has been no way to completely cure ONFH in clinical, except patients can only achieve the purpose of self‐care through total hip arthroplasty (THA).^2^, ^22^ Notably, alcoholism is the main factor of ONFH in the worldwide, especially for the adult patients.^23^ Therefore, it is urgent to discover and understand the mechanism of alcoholic ONFH, and develop new therapeutic drugs and methods for clinical usage.

After ethanol is absorbed into the body, it is oxidized into acetaldehyde in the mitochondria by the alcohol dehydrogenase; then acetaldehyde is further oxidized into non‐toxic acetate by the limited metabolized ALDH 2.^24^ What is more, both ethanol and acetaldehyde are toxic for many cells and organs leading to more than 60 diseases.^10^, ^25^ Since the expression level of ALDH 2 in the body is basically constant or difficult to change, so the key to the prevention and treatment of alcoholic diseases is to increase the activity of ALDH 2. Therefore, the ALDH 2 activators are to be highly promise drugs for pharmacotherapy for alcoholic diseases, Alda‐1 is a highly selective agonist of ALDH and capable of greatly elevating the ALDH 2 activity.^14^ A pile of reports has indicated the protective effect of Alda‐1 against numerous diseases via activating ALDH 2.

Accumulative previous studies have shown that obviously impaired osteogenesis capacity, the number and the osteogenic potential of osteoblasts of BMSCs in ONFH patients. Therefore, BMSCs are the key factor for the development and treatment of alcoholic ONFH. In the present study, ethanol significantly inhibited the proliferation and osteogenic differentiation of BMSCs, which was consistent with our previous research.^21^, ^26^ Moreover, Alda‐1 could obviously reverse the inhibition of ethanol‐induced proliferation and osteogenic differentiation of BMSCs. What is more, our work indicated that Alda‐1 alleviated the ethanol‐induced inhibitory osteogenesis of BMSCs by PI3K/AKT signalling pathway. PI3K/AKT signalling is a central pathway for the osteogenic differentiation of BMSCs. Alda‐1 accelerates cutaneous wound healing by Akt signalling pathway.^15^ Intriguingly, Alda‐1 significantly reduced the occurrence of ONFH and promoted osteogenic differentiation and new bone formation in the rat model of alcohol‐induced ONFH. Taken together, Alda‐1 might alleviate the ethanol‐induced ONFH by PI3K/AKT pathway via regulating the osteogenesis of BMSCs.

The vital pathological features of ONFH progress includes intramedullary fat deposition, increased number of adipocytes and enlarged lipid droplets.^27^, ^28^ It is well known that fat is mainly derived from adipogenic differentiation of BMSCs, and only a little of BMSCs would differentiate into adipocytes under normal conditions. Therefore, furthest inhibition the adipogenic capacity of BMSCs is the key point for preventing the development of ONFH. Accumulated studies indicate that ethanol triggers the transformation of BMSCs predominantly into adipocytes.^29^ Our results also demonstrated that ethanol significantly promoted the adipogenic differentiation, fortunately, Alda‐1 significantly retarded alcohol‐induced adipogenesis of BMSCs via AMPK pathway. Our previous studies indicated that ethanol promoted adipogenesis leading to ONFH by AMPK pathway.^21^ Moreover, Alda‐1 activation of ALDH 2 protected against ethanol‐induced cardiac defect and apoptosis via AMPK signalling.^30^ In summary, Alda‐1 might alleviate the ethanol‐induced ONFH via retarding the ethanol‐induced adipogenesis by AMPK pathway.

The femoral head is a highly vascularized tissue, blood vessels supply oxygen, minerals and nutrients for bone homoeostasis and remodelling, carry away the metabolites.^31^ Moreover, another name of ONFH is Aseptic ischaemic necrosis of the femoral head. Therefore, vascularization of the femoral heads is vital for prevention and treatment of ethanol‐induced ONFH. The previous research indicated that high dose of ethanol significantly inhibited the proliferation, migration and angiogenesis of HUVECs by terminally regulating growth factors (like VEGF and PDGF).^32^, ^33^ What is more, Alda‐1 activation of ALDH 2 increased angiogenesis via upregulation of VEGF receptors.^34^ Similarly, high dose of ethanol significantly inhibited the expression level of growth factors resulting in reduced angiogenesis, whereas Alda‐1 could rescue the inhibitory effect of ethanol on angiogenesis in the present study. Intriguingly, Alda‐1 significantly reduced the occurrence of ONFH and promoted vessel number and distribution in the rat model of alcohol‐induced ONFH.

In summary, Alda‐1 activation of ALDH 2 was highly demonstrated to protect ethanol‐induced ONFH by triggering new bone formation, reducing adipogenesis and stimulating vascularization. Therefore, ALDH 2 may be a potential therapeutic target, and small molecule Alda‐1 may be a promising pharmacotherapeutic for ONFH in the future.

## AUTHOR CONTRIBUTIONS

Hongping Yu and Xiaoyi Lin designed the study, analysed, interpreted the data and drafted the paper. Xiaoyi Lin, Daoming Zhu, Kaiyang Wang, Pengbo Luo, and Gang Rui participated in acquiring the data. Hongping Yu, Xiaoyi Lin, Daoming Zhu, Fuan Liu, and Youshui Gao designed the work and drafted the paper.

## CONFLICT OF INTEREST

The authors declare no conflict of interest.

## Supporting information


**TABLE S1** The RT‐PCR primers used in this study
**FIGURE S1** The average weight of each group rats was measured
**FIGURE S2** Effects of ethanol and Alda‐1 on the proliferation of BMSCs
**FIGURE S3** Effects of ethanol and Alda‐1 on the proliferation of HUVECs

## Data Availability

The datasets used in this study are available from corresponding authors on a reasonable request.
