# Supplementary material for: Activation of aldehyde dehydrogenase 2 protects ethanol‐induced osteonecrosis of the femoral head in rat model
Source: Cell Prolif. 2022 May 14;55(6):e13252. doi: 10.1111/cpr.13252 (PMC9201375; doi:10.1111/cpr.13252)
Supplement: Supplementary file 1 — TABLE S1 The RT‐PCR primers used in this study FIGURE S1 The average weight of each group rats was measured FIGURE S2 Effects of ethanol and Alda‐1 on the proliferation of BMSCs FIGURE S3 Effects of ethanol and Alda‐1 on the proliferation of HUVECs [file CPR-55-e13252-s001.docx]

**Supporting information**

**Activation of aldehyde dehydrogenase 2 protects ethanol-induced osteonecrosis of the femoral head in rat model**

Xiaoyi Lin^1^, Daoming Zhu^2^, Kaiyang Wang^3^, Pengbo Luo^4^, Gang Rui^1^, Youshui Gao^4*^, Fuan Liu^1*^, Hongping Yu^1*^.

1 Department of Orthopedic Surgery, The First Affiliated Hospital of Xiamen University, Xiamen, Fujian 361005, P.R. China.

2 Department of Medical Imaging, The Central Hospital of Enshi Tujia and Miao

Autonomous Prefecture, Enshi, Hubei, 445000, P.R. China

3 Department of Spine Surgery, Drum Tower Hospital of Nanjing University Medical School, 321, Zhongshan Rd, Nanjing, 210008, China

4 Department of Orthopedic Surgery, Shanghai Jiao Tong University Affiliated Sixth People’s Hospital, Shanghai 200233, China

Xiaoyi Lin and Daoming Zhu are co-first authors and contributed equally to this work.

Correspondence to:

Youshui Gao, Tel: 86-021-64369181, Fax: 86-21-64369181, E-mail: gaoyoushui@sjtu.edu.cn;

Fuan Liu, Tel: 86-13600937155, Fax: 86-592-2137327, E-mail: 13600937155@163.com

Hongping Yu, Tel: 86-13167055851, Fax: 86-592-2137327, Email: yuhp09@126.com

**Table S1**. The RT-PCR primers used in this study.

| Gene | Forward primers | Reverse primers |
| --- | --- | --- |
| GAPDH | 5’-CAGGTTGTCTCCTGCGACTT-3’ | 5’-TATGGGGGTCTGGGATGGAA-3’ |
| COL I | 5’-CTCAAGAAGTCCCTGCTCCTC-3’ | 5’-GACTGTCTTGCCCCAAGTTC-3’ |
| Runx 2 | 5’-GCATCCTTGGCTTTGCAGTC-3’ | 5’-AGTGTTTGCTGTAATGCGCC-3’ |
| OPN | 5’-CCGTTTAGGGCATGTGTTGC-3’ | 5’-CCGTCCATACTTTCGAGGCA-3’ |
| VEGF | 5’-CTGGGCTGTTCTCGCTT-3’ | 5’-CCCCTCTCCTCTTCCTTCT-3’ |
| PDGF | 5’-AGAGGACACGGGAAGGC-3’ | 5’-TGGCTGCTTTAGGTGGGT-3’ |
| EGF | 5’-TCAGTATGATACCTCACCCAGC-3’ | 5’-GGGTAGCCGTGTTCTCATGT-3’ |
| PPARγ | 5’-CCGCATTTTTCAAGGGTGCC-3’ | 5’-CCGCAGGCTTTTGAGGAACT-3’ |
| LPL | 5’-﻿CTGAGTGCTGGGAGCTTGAT-3’ | 5’-﻿GACGTGTGACAGAGGTACGG-3’ |
| Leptin | 5’-﻿CTGAGTGCTGGGAGCTTGAT-3’ | 5’-﻿GACGTGTGACAGAGGTACGG-3’ |


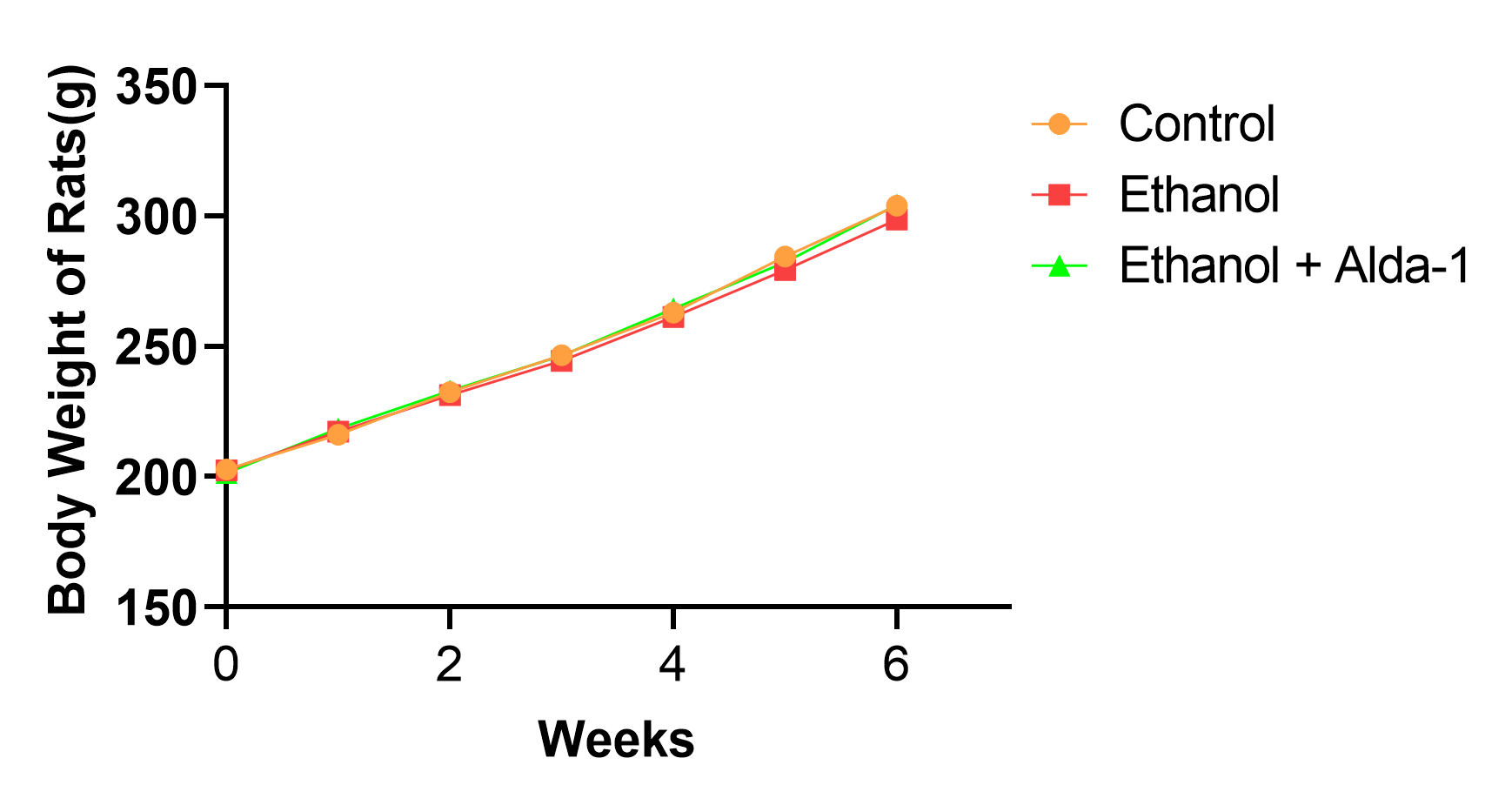


**Fig. S1** The average weight of each group rats was measured.


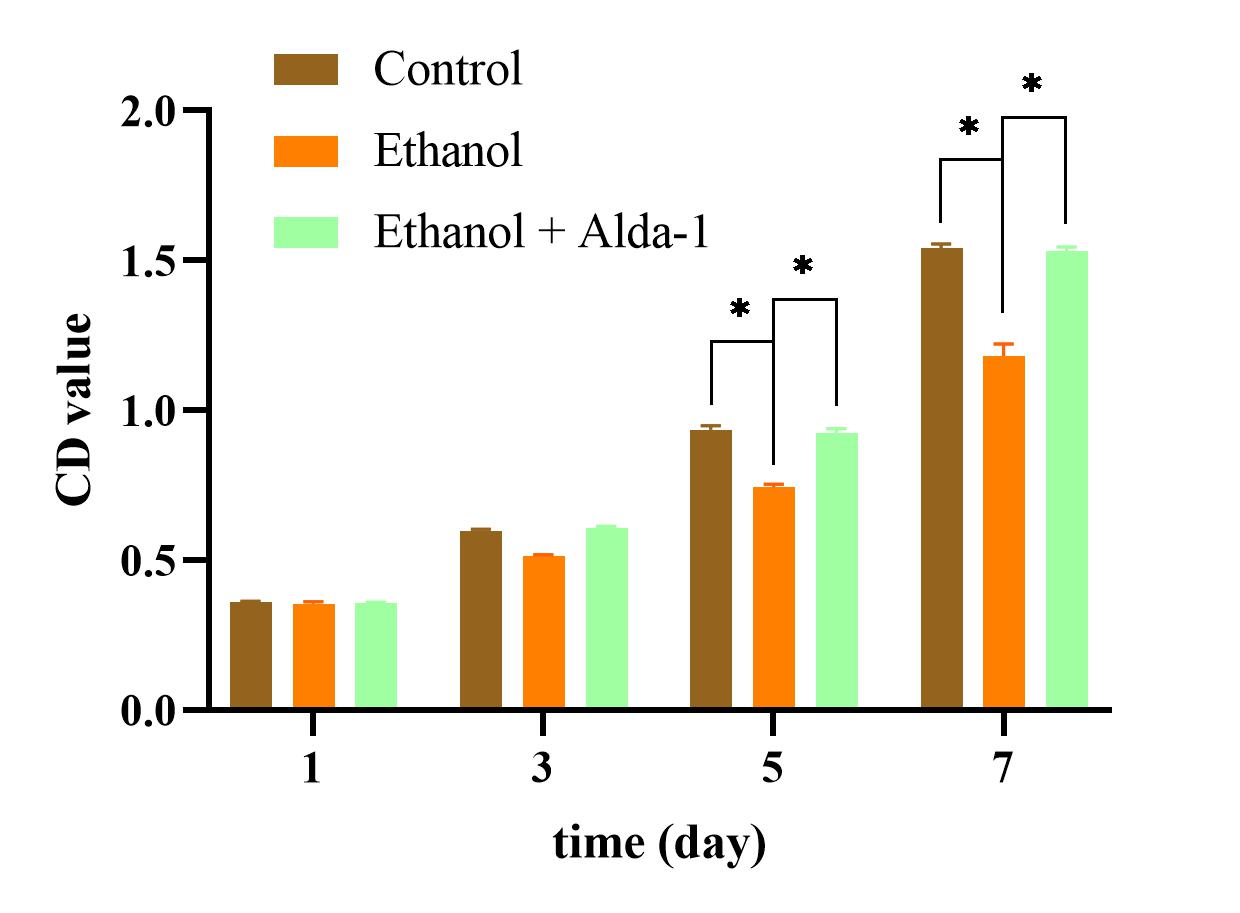


**Fig. S2** Effects of ethanol and Alda-1 on the proliferation of BMSCs.


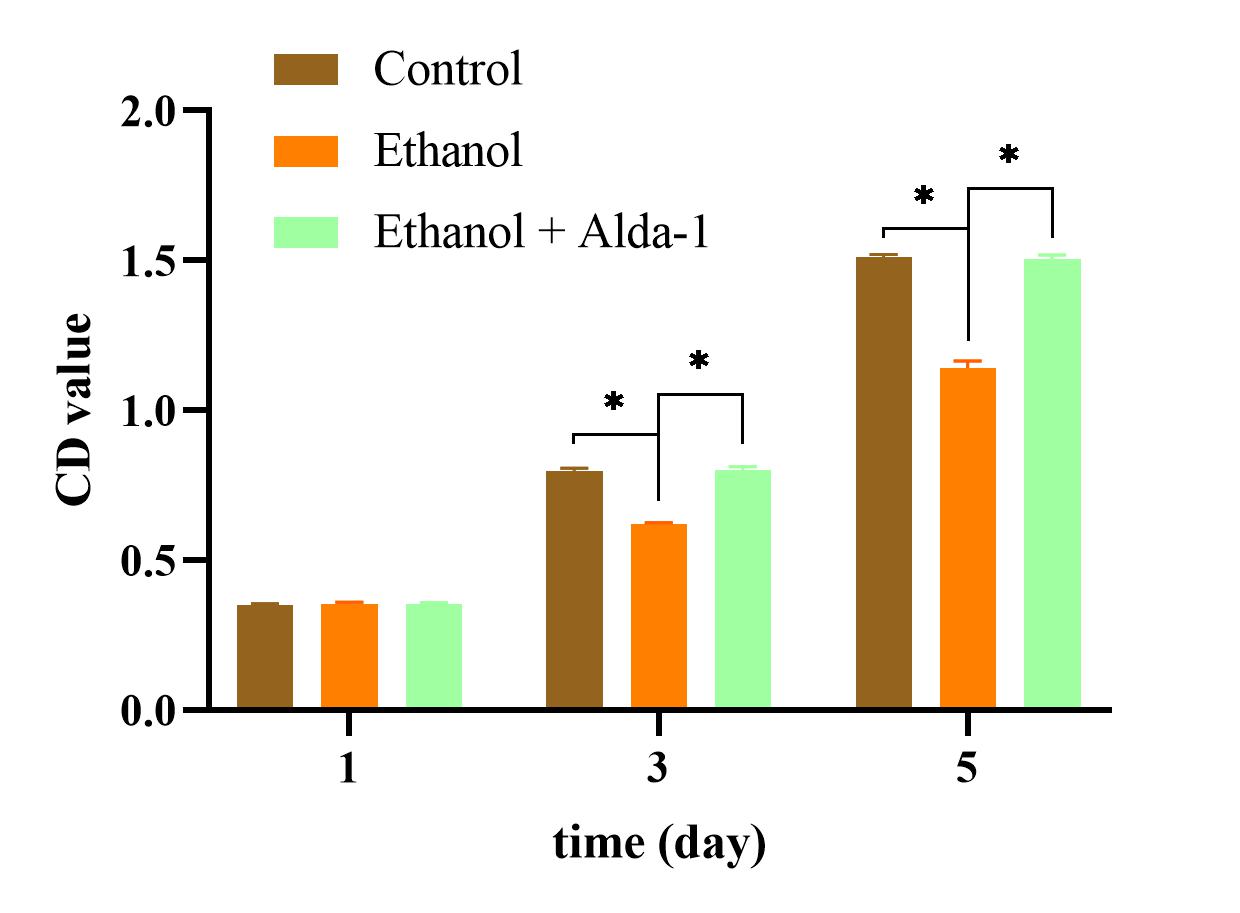


**Fig. S3** Effects of ethanol and Alda-1 on the proliferation of HUVECs.
